# (2*E*,4*R*,5*R*,6*S*)-2-(4,5,6-Trihy­droxy­cyclo­hex-2-en-1-yl­idene)acetonitrile

**DOI:** 10.1107/S1600536812035313

**Published:** 2012-08-23

**Authors:** Alphonsine N. Guedem, Louis P. Sandjo, Till Opatz, Dieter Schollmeyer, Bonaventure T. Ngadjui

**Affiliations:** a Department of Organic Chemistry, University of Yaounde I, PO Box 812 Yaounde, Cameroon; b University Mainz, Duesbergweg 10-14, 55128 Mainz, Germany

## Abstract

The crystal structure of the title compound, C_8_H_9_NO_3_, is characterized by a complex three-dimensional hydrogen-bond network in which every mol­ecule is connected to six symmetry-related neighbours.

## Related literature

For the isolation of this natural product, see: Hua *et al.* (2004 ▶). For previous phytochemical and biological studies of the stem bark of *Thecacoris annobonae*, see: Kuete *et al.* (2010 ▶).
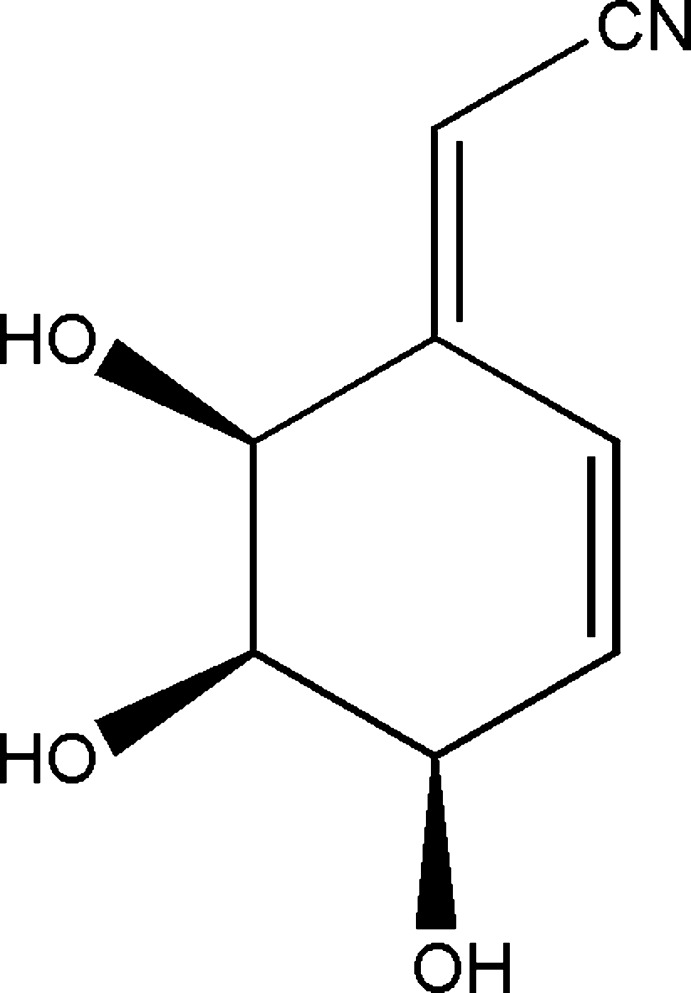


## Experimental

### 

#### Crystal data

C_8_H_9_NO_3_
*M* *_r_* = 167.16
Monoclinic, 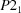

*a* = 4.8159 (5) Å
*b* = 10.2482 (5) Å
*c* = 8.3573 (9) Å
β = 102.842 (4)°
*V* = 402.15 (6) Å^3^
*Z* = 2
Cu *K*α radiation
μ = 0.90 mm^−1^
*T* = 193 K
0.60 × 0.06 × 0.06 mm

#### Data collection

Enraf–Nonius CAD-4 diffractometer
2174 measured reflections
1514 independent reflections
1501 reflections with *I* > 2σ(*I*)
*R*_int_ = 0.021
3 standard reflections every 60 min intensity decay: 5%

#### Refinement

*R*[*F* ^2^ > 2σ(*F* ^2^)] = 0.026
*wR*(*F* ^2^) = 0.071
*S* = 1.08
1514 reflections
146 parameters
1 restraint
All H-atom parameters refined
Δρ_max_ = 0.21 e Å^−3^
Δρ_min_ = −0.12 e Å^−3^
Absolute structure: Flack, (1983 ▶)
Flack parameter: −0.04 (16)

### 

Data collection: *CAD-4 Software* (Enraf–Nonius, 1989 ▶); cell refinement: *CAD-4 Software*; data reduction: *CORINC* (Dräger & Gattow, 1971 ▶); program(s) used to solve structure: *SIR97* (Altomare *et al.*, 1999 ▶); program(s) used to refine structure: *SHELXL97* (Sheldrick, 2008 ▶); molecular graphics: *PLATON* (Spek, 2009 ▶); software used to prepare material for publication: *PLATON*.

## Supplementary Material

Crystal structure: contains datablock(s) I, global. DOI: 10.1107/S1600536812035313/nc2287sup1.cif

Structure factors: contains datablock(s) I. DOI: 10.1107/S1600536812035313/nc2287Isup2.hkl

Supplementary material file. DOI: 10.1107/S1600536812035313/nc2287Isup3.cml

Additional supplementary materials:  crystallographic information; 3D view; checkCIF report

## Figures and Tables

**Table 1:** Hydrogen-bond geometry (Å, °)

*D*—H⋯*A*	*D*—H	H⋯*A*	*D*⋯*A*	*D*—H⋯*A*
O7—H7⋯O9^i^	0.79 (2)	1.93 (2)	2.6885 (14)	160 (2)
O8—H8⋯N12^ii^	0.77 (2)	2.18 (2)	2.9138 (16)	160 (2)
O9—H9⋯O7^iii^	0.78 (3)	2.02 (2)	2.7944 (15)	170 (2)

Symmetry codes: (i) 
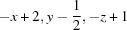
; (ii) 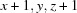; (iii) 
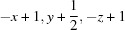
.

**Fig. 1.**
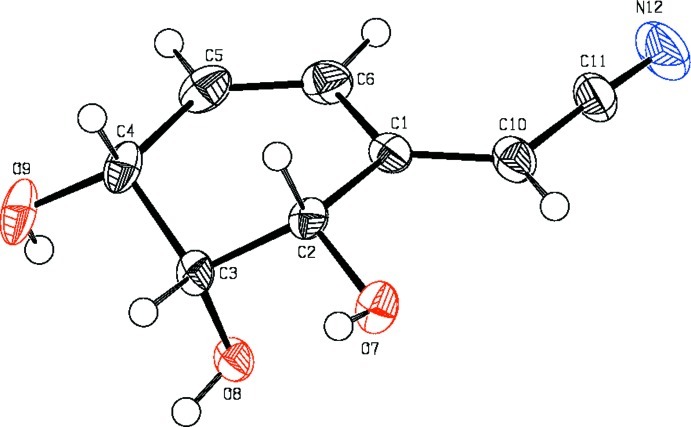
Crystal structure of the title compound with labelling and displacement ellipsoids drawn at the 50% probability level.

**Fig. 2.**
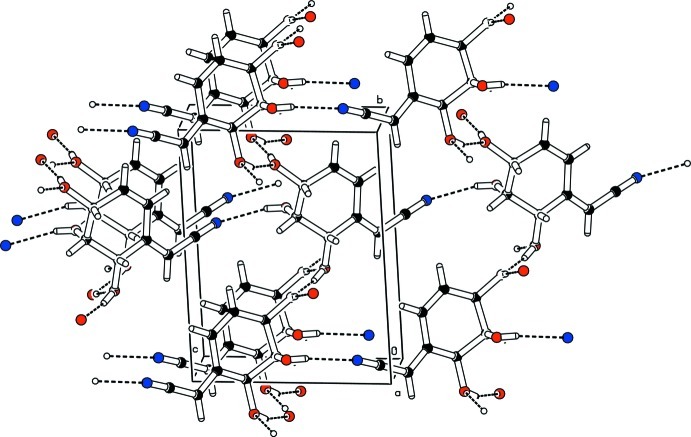
Crystal structure of the title compound with view along the a-axis. For calrity only H-atoms involved in hydrogen bonds are shown. Intermolecular hydrogen bonding is represented as dashed lines.

## supplementary crystallographic information

### Comment

Previous phytochemical and biological studies of the stem bark of Thecacoris annobonae (Euphorbiaceae) led to several bioactive secondary metabolites: Kuete *et al.* (2010). In the continuation of this investigation, the title compound was isolated from the leaves of the same plant using chromatographic methods and characterized by single crystal X-ray diffraction. It should be noted that this natural product was previously obtained from the root of Semiaquilegia adoxoides (Ranunculaceae): Hua *et al.* (2004).

In the crystal structure of the title compound the six membered ring adopts an envelope conformation in which C(3) is 0.678 (1) Å below the ring plane (Fig. 1). The acrylonitrile group is nearly coplanar to the least square plane of the ring system. The packing is characterized by a complex three-dimensional network formed by hydrogen bonds. Every molecule interacts by hydrogen bonds with six symmetry related molecules. While the hydroxyl groups O7 and O9 are both donor and acceptor of hydrogen bonds, O8 only interacts with N12 *via* hydrogen bonding (Fig. 2 and Table 1).

### Experimental

Air-dried powder of leaves of Thecacoris annobonae (1.37 kg) was successively macerated with hexane, ethyl acetate and methanol for two days each. Three fractions H (30 g), E (45 g), and *M* (61 g) were collected. The Methanol fraction *M* was subjected to a silica gel column chromatography eluted with CH_2_Cl_2_ to MeOH gradient yielding 7 mg of this secondary metabolite. It crystallized as needles in three of the fractions eluted with the mixture CH_2_Cl_2_/MeOH in a ratio of 97:3.

### Refinement

All hydrogen atoms were located from a difference Fourier map and refined with isotropic displacement parameters. The absolute structure was determined on the basis of 705 Friedel pairs.

### Crystal data

**Table:**

C_8_H_9_NO_3_	*F*(000) = 176
*M**_r_* = 167.16	*D*_x_ = 1.380 Mg m^−^^3^
Monoclinic, *P*2_1_	Cu *K*α radiation, λ = 1.54178 Å
Hall symbol: P 2yb	Cell parameters from 25 reflections
*a* = 4.8159 (5) Å	θ = 35–46°
*b* = 10.2482 (5) Å	µ = 0.90 mm^−^^1^
*c* = 8.3573 (9) Å	*T* = 193 K
β = 102.842 (4)°	Needle, colourless
*V* = 402.15 (6) Å^3^	0.60 × 0.06 × 0.06 mm
*Z* = 2	

### Data collection

**Table:**

Enraf–Nonius CAD-4 diffractometer	*R*_int_ = 0.021
Radiation source: rotating anode	θ_max_ = 70.0°, θ_min_ = 5.4°
Graphite monochromator	*h* = −5→5
ω/2θ scans	*k* = −12→12
2174 measured reflections	*l* = −10→10
1514 independent reflections	3 standard reflections every 60 min
1501 reflections with *I* > 2σ(*I*)	intensity decay: 5%

### Refinement

**Table:**

Refinement on *F*^2^	Hydrogen site location: inferred from neighbouring sites
Least-squares matrix: full	All H-atom parameters refined
*R*[*F*^2^ > 2σ(*F*^2^)] = 0.026	*w* = 1/[σ^2^(*F*_o_^2^) + (0.0474*P*)^2^ + 0.0306*P*] where *P* = (*F*_o_^2^ + 2*F*_c_^2^)/3
*wR*(*F*^2^) = 0.071	(Δ/σ)_max_ < 0.001
*S* = 1.08	Δρ_max_ = 0.21 e Å^−^^3^
1514 reflections	Δρ_min_ = −0.12 e Å^−^^3^
146 parameters	Extinction correction: *SHELXL97* (Sheldrick, 2008), Fc^*^=kFc[1+0.001xFc^2^λ^3^/sin(2θ)]^-1/4^
1 restraint	Extinction coefficient: 0.018 (3)
Primary atom site location: structure-invariant direct methods	Absolute structure: Flack, (1983)
Secondary atom site location: difference Fourier map	Flack parameter: −0.04 (16)

### Special details

**Table:**

Experimental. ^1^H–, ^13^C– and two-dimensional-NMR spectra were recorded on Bruker AVANCE II-400 MHz s pectrometer equipped with a 5 mm observe probe and a *z*-gradient coil using standard pulse sequences. HR-ESI-MS was carried out on a Waters Q-TOF Ultima III mass spectrometer. HR-ESI-MS m/*z* 190.0470 (calcd. for [C_8_H_9_NO_3_+Na]^+^ 190.0475); NMR (^1^H-NMR, 400 MHz, acetone-d_6_): 6.55 (1*H*, dd, J = 2.5, 10.1 Hz, H-2), 6.04 (1*H*, dd, J = 1.8, 10.1 Hz, H-3), 4.11–4.16 (1*H*, m, H-4), 4.40–4.44 (1*H*, m, H-5), 4.44–4.50 (1*H*, m, H-6), 5.63 (1*H*, s, H-7), 4.60 (1*H*, d, J = 7.9 Hz, OH-5), 4.16–4.18 (2*H*, m, OH-4 and 6); (^13^C-NMR, 100 MHz, acetone-d_6_): 159.7 (C-1), 124.0 (C-2), 139.6 (C-3), 74.5 (C-4), 69.6 (C-5), 71.9 (C-6),93.7 (C-7), 117.6 (C-8).
Geometry. All e.s.d.'s (except the e.s.d. in the dihedral angle between two l.s. planes) are estimated using the full covariance matrix. The cell e.s.d.'s are taken into account individually in the estimation of e.s.d.'s in distances, angles and torsion angles; correlations between e.s.d.'s in cell parameters are only used when they are defined by crystal symmetry. An approximate (isotropic) treatment of cell e.s.d.'s is used for estimating e.s.d.'s involving l.s. planes.
Refinement. Refinement of *F*^2^ against ALL reflections. The weighted *R*-factor *wR* and goodness of fit *S* are based on *F*^2^, conventional *R*-factors *R* are based on *F*, with *F* set to zero for negative *F*^2^. The threshold expression of *F*^2^ > σ(*F*^2^) is used only for calculating *R*-factors(gt) *etc*. and is not relevant to the choice of reflections for refinement. *R*-factors based on *F*^2^ are statistically about twice as large as those based on *F*, and *R*- factors based on ALL data will be even larger.

### Fractional atomic coordinates and isotropic or equivalent isotropic displacement parameters (Å^2^)

**Table:**

	*x*	*y*	*z*	*U*_iso_*/*U*_eq_	
C1	0.4167 (3)	0.60278 (13)	0.16499 (15)	0.0204 (3)	
C2	0.6543 (3)	0.54538 (12)	0.29376 (15)	0.0193 (3)	
H2	0.822 (3)	0.5471 (15)	0.2490 (18)	0.014 (3)*	
C3	0.7220 (3)	0.63193 (13)	0.44623 (15)	0.0195 (3)	
H3	0.881 (4)	0.5923 (17)	0.525 (2)	0.023 (4)*	
C4	0.8147 (3)	0.76563 (13)	0.39626 (17)	0.0244 (3)	
H4	0.992 (4)	0.7516 (17)	0.364 (2)	0.023 (4)*	
C5	0.6001 (3)	0.81945 (14)	0.2530 (2)	0.0291 (3)	
H5	0.591 (4)	0.911 (2)	0.238 (2)	0.041 (5)*	
C6	0.4214 (3)	0.74407 (14)	0.14727 (17)	0.0277 (3)	
H6	0.289 (4)	0.7814 (18)	0.059 (2)	0.023 (4)*	
O7	0.5884 (2)	0.41531 (9)	0.32843 (12)	0.0246 (2)	
H7	0.730 (5)	0.381 (2)	0.375 (2)	0.038 (5)*	
O8	0.47685 (18)	0.64281 (10)	0.51340 (11)	0.0221 (2)	
H8	0.530 (4)	0.644 (2)	0.607 (3)	0.031 (5)*	
O9	0.8700 (2)	0.85292 (10)	0.53125 (14)	0.0320 (3)	
H9	0.730 (5)	0.870 (2)	0.559 (3)	0.045 (6)*	
C10	0.2197 (3)	0.52459 (15)	0.07211 (16)	0.0252 (3)	
H10	0.220 (4)	0.431 (2)	0.0850 (19)	0.024 (4)*	
C11	−0.0065 (3)	0.57476 (17)	−0.05275 (17)	0.0316 (3)	
N12	−0.1938 (3)	0.61195 (18)	−0.15135 (17)	0.0451 (4)	

### Atomic displacement parameters (Å^2^)

**Table:**

	*U*^11^	*U*^22^	*U*^33^	*U*^12^	*U*^13^	*U*^23^
C1	0.0145 (6)	0.0289 (7)	0.0186 (5)	0.0032 (5)	0.0053 (4)	0.0007 (5)
C2	0.0106 (6)	0.0241 (6)	0.0233 (6)	0.0012 (5)	0.0043 (5)	0.0010 (5)
C3	0.0086 (5)	0.0255 (6)	0.0227 (5)	0.0031 (4)	−0.0001 (4)	0.0005 (5)
C4	0.0128 (6)	0.0260 (7)	0.0344 (7)	−0.0030 (5)	0.0054 (5)	−0.0046 (5)
C5	0.0271 (8)	0.0222 (7)	0.0391 (8)	−0.0003 (5)	0.0098 (6)	0.0065 (5)
C6	0.0229 (7)	0.0318 (8)	0.0273 (6)	0.0039 (6)	0.0034 (5)	0.0097 (6)
O7	0.0167 (5)	0.0204 (5)	0.0340 (5)	0.0034 (4)	−0.0001 (4)	0.0022 (4)
O8	0.0134 (4)	0.0328 (5)	0.0197 (4)	0.0006 (4)	0.0030 (3)	−0.0018 (4)
O9	0.0135 (5)	0.0332 (6)	0.0489 (6)	−0.0054 (4)	0.0060 (4)	−0.0162 (5)
C10	0.0186 (6)	0.0354 (7)	0.0212 (6)	0.0016 (5)	0.0034 (5)	−0.0014 (5)
C11	0.0250 (7)	0.0477 (9)	0.0208 (6)	−0.0052 (6)	0.0024 (6)	−0.0037 (6)
N12	0.0319 (7)	0.0705 (11)	0.0269 (6)	0.0011 (7)	−0.0064 (5)	0.0055 (6)

### Geometric parameters (Å, º)

**Table:**

C1—C10	1.3479 (19)	C4—H4	0.963 (18)
C1—C6	1.4563 (19)	C5—C6	1.334 (2)
C1—C2	1.5054 (16)	C5—H5	0.94 (2)
C2—O7	1.4151 (15)	C6—H6	0.943 (18)
C2—C3	1.5272 (17)	O7—H7	0.79 (2)
C2—H2	0.962 (16)	O8—H8	0.77 (2)
C3—O8	1.4200 (16)	O9—H9	0.78 (3)
C3—C4	1.5277 (18)	C10—C11	1.426 (2)
C3—H3	0.981 (17)	C10—H10	0.96 (2)
C4—O9	1.4178 (17)	C11—N12	1.144 (2)
C4—C5	1.5019 (19)		
			
C10—C1—C6	123.87 (12)	C5—C4—C3	110.86 (10)
C10—C1—C2	120.34 (12)	O9—C4—H4	107.3 (10)
C6—C1—C2	115.77 (11)	C5—C4—H4	109.1 (10)
O7—C2—C1	110.18 (11)	C3—C4—H4	105.9 (10)
O7—C2—C3	113.10 (10)	C6—C5—C4	122.94 (13)
C1—C2—C3	110.92 (10)	C6—C5—H5	119.0 (12)
O7—C2—H2	110.0 (10)	C4—C5—H5	118.1 (12)
C1—C2—H2	106.6 (9)	C5—C6—C1	122.05 (12)
C3—C2—H2	105.8 (9)	C5—C6—H6	120.6 (11)
O8—C3—C2	109.47 (10)	C1—C6—H6	117.4 (11)
O8—C3—C4	110.90 (11)	C2—O7—H7	108.3 (16)
C2—C3—C4	108.30 (10)	C3—O8—H8	106.5 (14)
O8—C3—H3	111.0 (10)	C4—O9—H9	111.1 (17)
C2—C3—H3	108.2 (10)	C1—C10—C11	122.16 (14)
C4—C3—H3	108.9 (10)	C1—C10—H10	123.0 (10)
O9—C4—C5	112.14 (12)	C11—C10—H10	114.9 (10)
O9—C4—C3	111.28 (11)	N12—C11—C10	177.70 (18)
			
C10—C1—C2—O7	−16.29 (16)	O8—C3—C4—C5	−68.05 (13)
C6—C1—C2—O7	165.07 (11)	C2—C3—C4—C5	52.09 (14)
C10—C1—C2—C3	−142.30 (13)	O9—C4—C5—C6	−149.07 (14)
C6—C1—C2—C3	39.06 (15)	C3—C4—C5—C6	−24.01 (19)
O7—C2—C3—O8	−63.88 (13)	C4—C5—C6—C1	1.4 (2)
C1—C2—C3—O8	60.49 (13)	C10—C1—C6—C5	172.38 (14)
O7—C2—C3—C4	175.09 (9)	C2—C1—C6—C5	−9.0 (2)
C1—C2—C3—C4	−60.54 (13)	C6—C1—C10—C11	−0.6 (2)
O8—C3—C4—O9	57.48 (13)	C2—C1—C10—C11	−179.10 (12)
C2—C3—C4—O9	177.62 (10)	C1—C10—C11—N12	−141 (4)

### Hydrogen-bond geometry (Å, º)

**Table:**

*D*—H···*A*	*D*—H	H···*A*	*D*···*A*	*D*—H···*A*
O7—H7···O9^i^	0.79 (2)	1.93 (2)	2.6885 (14)	160 (2)
O8—H8···N12^ii^	0.77 (2)	2.18 (2)	2.9138 (16)	160 (2)
O9—H9···O7^iii^	0.78 (3)	2.02 (2)	2.7944 (15)	170 (2)

Symmetry codes: (i) −*x*+2, *y*−1/2, −*z*+1; (ii) *x*+1, *y*, *z*+1; (iii) −*x*+1, *y*+1/2, −*z*+1.
